# Abiotic Racemization Kinetics of Amino Acids in Marine Sediments

**DOI:** 10.1371/journal.pone.0071648

**Published:** 2013-08-12

**Authors:** Andrew D. Steen, Bo Barker Jørgensen, Bente Aa. Lomstein

**Affiliations:** 1 Center for Geomicrobiology, Aarhus University, Aarhus, Denmark; 2 Department of Bioscience, Aarhus University, Aarhus, Denmark; University of California, Merced, United States of America

## Abstract

The ratios of d- versus l-amino acids can be used to infer the sources and composition of sedimentary organic matter. Such inferences, however, rely on knowing the rates at which amino acids in sedimentary organic matter racemize abiotically between the d- and the l-forms. Based on a heating experiment, we report kinetic parameters for racemization of aspartic acid, glutamic acid, serine, and alanine in bulk sediment from Aarhus Bay, Denmark, taken from the surface, 30 cm, and 340 cm depth below seafloor. Extrapolation to a typical cold deep sea sediment temperature of 3°C suggests racemization rate constants of 0.50×10^−5^–11×10^−5^ yr^−1^. These results can be used in conjunction with measurements of sediment age to predict the ratio of d:l amino acids due solely to abiotic racemization of the source material, deviations from which can indicate the abundance and turnover of active microbial populations.

## Introduction

Most biologically-produced amino acids are in the l- stereochemical conformation, however bacterial cell walls also contain certain d-amino acids [1]. d-amino acids can also be present in other bacterial structures such as teichoic acids, lipopolysaccharides, polypeptides, lipopetides, siderophores, and as free d-amino acids [2] and even occur in some archaea [3]. Because the sources of d-amino acids can be well constrained, d:l amino acid ratios can be used to estimate the contribution of bacterially-sourced organic matter to the total organic pool in the water column [2], [4], [5] and in sediments [6], [7], [8]. When combined with other biomarkers, d:l ratios can also be used to model carbon oxidation rates as well as turnover times of bacterial biomass and necromass (dead biomass) [9], [10]. All of these models rely, explicitly or implicitly, on knowledge of the rate constant for the abiotic racemization (interconversion between D- and L- forms) of amino acids in natural organic matter. At typical environmental temperature of less than 30°C, these racemization rate constants are slow enough that racemization half-lives (the time it takes for d:l ratios to proceed halfway to equilibrium) are on the order of thousands to hundreds of thousands of years [11].

When abiotic racemization rates have been used as a parameter in models concerning organic matter in marine systems, the rate constants used are typically taken from Bada [12], who measured rates using free amino acids in aqueous solution. Racemization rate constants are driven by the stability of the carboanion intermediate [13], however, which is influenced by a range of factors including pH, the presence of chemical stabilizers [14] or other electron-withdrawing groups, incorporation of amino acids into geomacromolecules [15], and other factors. All these factors may differ for amino acids bound in macromolecules in sedimentary organic matter. Due to these factors, racemization rate constants may differ among similar materials, such as the bulk tissue of related invertebrates or different organs of the same species [16].

Here we report the results of a heating experiment to measure racemization rate constants and Arrhenius parameters (activation energy *E_a_* and frequency factor *A*, eq. 1) for the amino acids aspartic acid (Asx), glutamic acid (Glx), serine (Ser) and alanine (Ala) in bulk sedimentary organic matter, in the absence of microbial growth. These four amino acids are the most frequently measured in sediments [17]. Activation energy represents the energy difference between reactants and the transition state, and indicates the temperature sensitivity of the reaction rate: reaction rates change more as a function of temperature for reactions with large *E_a_* than reactions with small *E_a_*. For racemization reactions, the frequency factor is essentially a scaling constant. We also rigorously estimate the error involved in extrapolating from rate constants measured at high temperatures (at which racemization occurs on laboratory-measurable timescale) to environmental temperatures, at which racemization rate constants are 3–6 orders of magnitude lower. The measurements presented here will allow more accurate use of enantiomeric ratios of amino acids in bulk sediments as indicators of bacterial necromass and carbon turnover.

## Materials and Methods

### Samples and Experimental Setup

Sediment was collected from Station M1 (56°07.066 N, 10°20.793 E), in central Aarhus Bay, Denmark, and stored anoxically prior to processing. The water depth is 16 m, and the water column is stratified during most of the year, with a surface layer derived from the Baltic Sea at 15–25 salinity overlying a bottom layer derived from the Kattegat at 29–33 salinity. Hypoxia may occur in the bottom water in the spring through fall. Bottom water temperatures may vary between 0–15°C with an annual mean of 7°C. A site description is given by Jørgensen [18]. No special permission was required to sample Station M1, and sampling did not harm any endangered species.

Rumohr-Lot core sections from 0–10 cm depth below seafloor (‘surface’) and 20–40 cm depth below seafloor (‘30 cm’) and a gravity core section from 335–350 cm (‘340 cm’) were homogenized using a mortar and pestle. Wet sediment (2 g) was dispensed into 5-ml gastight glass vials, which were subsequently purged with N_2_ and stored at 4°C. Based on ^14^C dating of the deeper sediment (J. B. Jensen, unpublished data), the ages since deposition of the sediment samples were approximately 25, 150, and 5,000 yr, respectively.

Because abiotic racemization rates are too slow to measure at the *in situ* temperatures on a human timescale, racemization incubations were carried out in temperature-controlled ovens at 105°C (for up to 168 hours), 77°C (up to 602 hours), 58.5°C (up to 165 days) and 49.5°C (up to 165 days). The air temperature in each oven (except the 105°C oven, which has high-precision temperature control) was monitored using temperature loggers and was in all cases stable to within ±0.5°C. At each of 12 timepoints, one sediment vial from each depth was removed from the oven, cooled immediately in an ice bath, and then stored at −20°C prior to preparation for HPLC analysis.

To prevent growth of microorganisms during the incubations, sediments were sterilized prior to incubation by autoclaving three times for 5 minutes each, with 48 hours at room temperature between autoclavings. The maximum autoclave temperature was 121°C. Multiple autoclavings were necessary to kill thermophilic spores, which are widespread in cold marine sediment [19]. According to the temperature coefficients for racemization determined from this study the autoclaving was too short to generate detectable changes in d:l amino acid ratios.

### HPLC Analysis

Amino acid concentrations and d:l ratios were determined as described by Lomstein et al. [17] with a modification to the HPLC gradient as described below. Briefly, samples were lyophilized, and approximately 0.25 g dry sediment was hydrolyzed in 6 N HCl under N_2_ for exactly 24 hours at 105°C. Hydrolysate (100 µl) was dried under vacuum at approx 40°C, redissolved in 100 µl Milli-Q water, and dried again. Hydrolysate was then redissolved in 4 ml Milli-Q water, and amino acid enantiomers were derivatized with *o*-phthaldialdehyde (OPA) and separated via HPLC [20]. In contrast to Lomstein et al [17], the strong mobile phase contained 80% methanol : 20% H_2_O, rather than 100% methanol, which markedly improved the separation between l- and d-Asx. All measurements primarily reflect the concentrations of combined amino acids, since dissolved free amino acids make a negligible (<0.1%) contribution to total amino acid concentrations in sediments [7].

### Data Analysis

Racemization rate constants, *k,* are given by
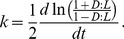
(1)where d and l indicate the concentrations of the d- and l- isomer, respectively [21]. Rate constants are related to temperature by the Arrhenius equation
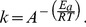
(2)in which R is the universal gas constant and T is temperature. Racemization half-life (the amount of time required for a mixture to proceed halfway to racemic equilibrium) is calculated as



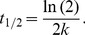
(3)
[21].

Calculation of the relationship between racemization rate constants and temperature from a heating experiment as described here requires two sequential functional fits to data. First, the transformed d:l values must be fit to incubation time in order to determine the racemization rate constant at each temperature. Second, in order to predict rate constants at environmentally relevant temperatures, data must be extrapolated over 3 or more orders of magnitude relative to observed rate constants. This large extrapolation has the potential to introduce substantial error, even if the underlying fits to data qualitatively appear to be good. In order to estimate the error associated with these fits and extrapolations, we employed a bootstrap resampling approach [22]. This approach has the significant advantage over parametric error estimation techniques that it does not rely on any assumptions about the distribution of error in the underlying data. Ensembles of 10^5^ artificial datasets were created by randomly resampling the dataset of d:l values versus time for each incubation. E_a_ and frequency factors A were calculated for each synthetic data set, and then rate constants from −4°C to 105°C were calculated for each synthetic data set according to equation 2. Median, 15.9 percentile, and 84.1 percentile (corresponding to +/− one standard deviation of a normal distribution) of E_a_ and A, and of each predicted rate constant, *k*, are reported as estimates of the predicted value and error for each parameter. In order to validate the bootstrap predictions, E_a_ and A were also predicted using the more traditional method of linear least squares fitting of the original data.

Kaiser and Benner [23] quantified the artifact induced by racemization during the acid hydrolysis procedure, and described this racemization in terms of the kinetic model of Equation 1. According to this model acid hydrolysis is expected to increase ln[(1+d:l)/(1−d:l)] by the same amount in each sample (for brevity, we will refer to the quantity ln[(1+d:l)/(1−d:l)] throughout the manuscript as ‘transformed d:l’). Since our analysis starts with determining the rate of change of transformed d:l as a function of time during incubations, hydrolysis-induced racemization does not affect the rate constants we calculate.

## Results and Discussion

### Abiotic Racemization Rate Constants and Arrhenius Parameters

**Figure 1:**
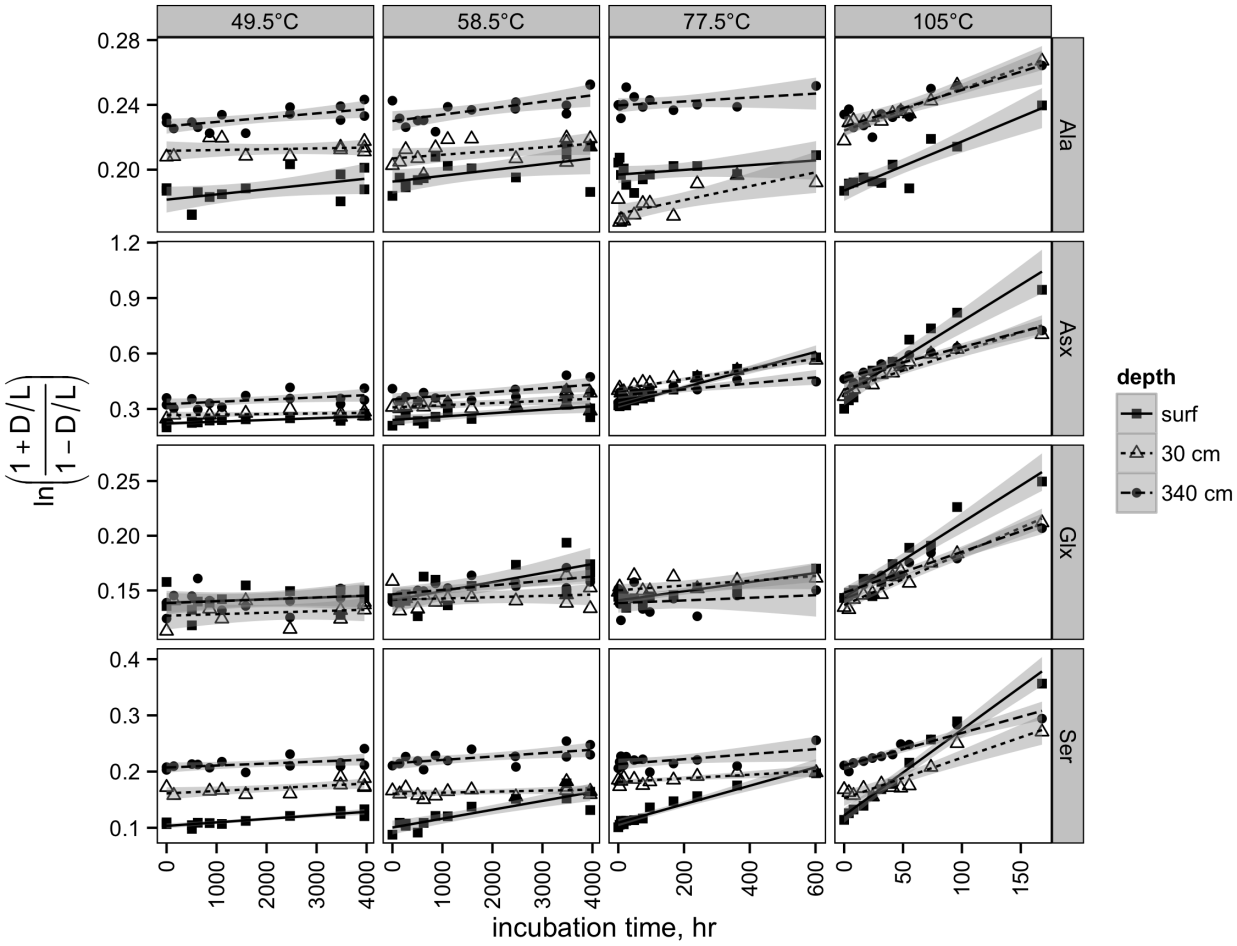
Racemization of aspartic acid+asparagine (Asx), glutamic acid+glutamine (Glx), serine (Ser) and alanine (Ala). amino acids in sediment from three different depths measured at four different temperatures as a function of time.

Raw D:L values observed in the heating experiment are listed in table S1, and observed racemization rate constants at elevated temperature are listed in Table 1. In general, the plot of transformed d:l vs. incubation time was linear within analytical error in agreement with equation 1 (Fig. 1). Only Asx in surface and medium-depth sediments incubated at 105°C, and Glx in surface sediments at 105°C had slightly curved plots of transformed d:l vs. incubation time. The curvature may be due to the fact that the observed racemization rates for Asx and Glx represent the sum of racemization rates of aspartic acid and asparagine, and glutamic acid and glutamine, respectively, because the hydrolysis step deaminates asparagine (Asn) to aspartic acid (Asp) and glutamine (Gln) to glutamic acid (Glu). Asn and Gln racemize faster than Asp and Glu due to the electron-withdrawing capacity of the secondary amine groups [24]. This effect can lead to a curved relationship between transformed d:l of Asx and Glx, and time [25].

**Table 1:** Observed racemization rate constants, yr^−1^.

		105°C	77°C	58.5°C	49.5°C
Asx	Surf	17.3±1.8	2.10±0.14	0.081±0.025	0.0421±0.0092
	30 cm	9.06±0.84	1.21±0.10	0.051±0.024	0.014±0.013
	340 cm	7.22±0.60	0.718±0.16	0.089±0.030	0.053±0.026
Glx	Surf	2.98±0.26	0.182±0.033	0.036±0.011	0.0068±0.0095
	30 cm	2.06±0.14	0.0953±0.047	0.0051±0.0075	0.0061±0.0089
	340 cm	1.64±0.14	0.0523±0.078	0.018±0.005	0.0075±0.0081
Ser	Surf	6.68±0.38	0.718±0.057	0.069±0.011	0.0275±0.0039
	30 cm	3.17±0.40	0.142±0.039	0.0081±0.0063	0.0192±0.0077
	340 cm	2.53±0.25	0.196±0.087	0.0264±0.0098	0.0155±0.0079
Ala	Surf	1.33±0.18	0.0639±0.047	0.016±0.007	0.0142±0.0070
	30 cm	1.13±0.09	0.187±0.050	0.0100±0.0053	0.0022±0.0041
	340 cm	0.985±0.18	0.0530±0.039	0.0174±0.0054	0.0116±0.0040

**Figure 2:**
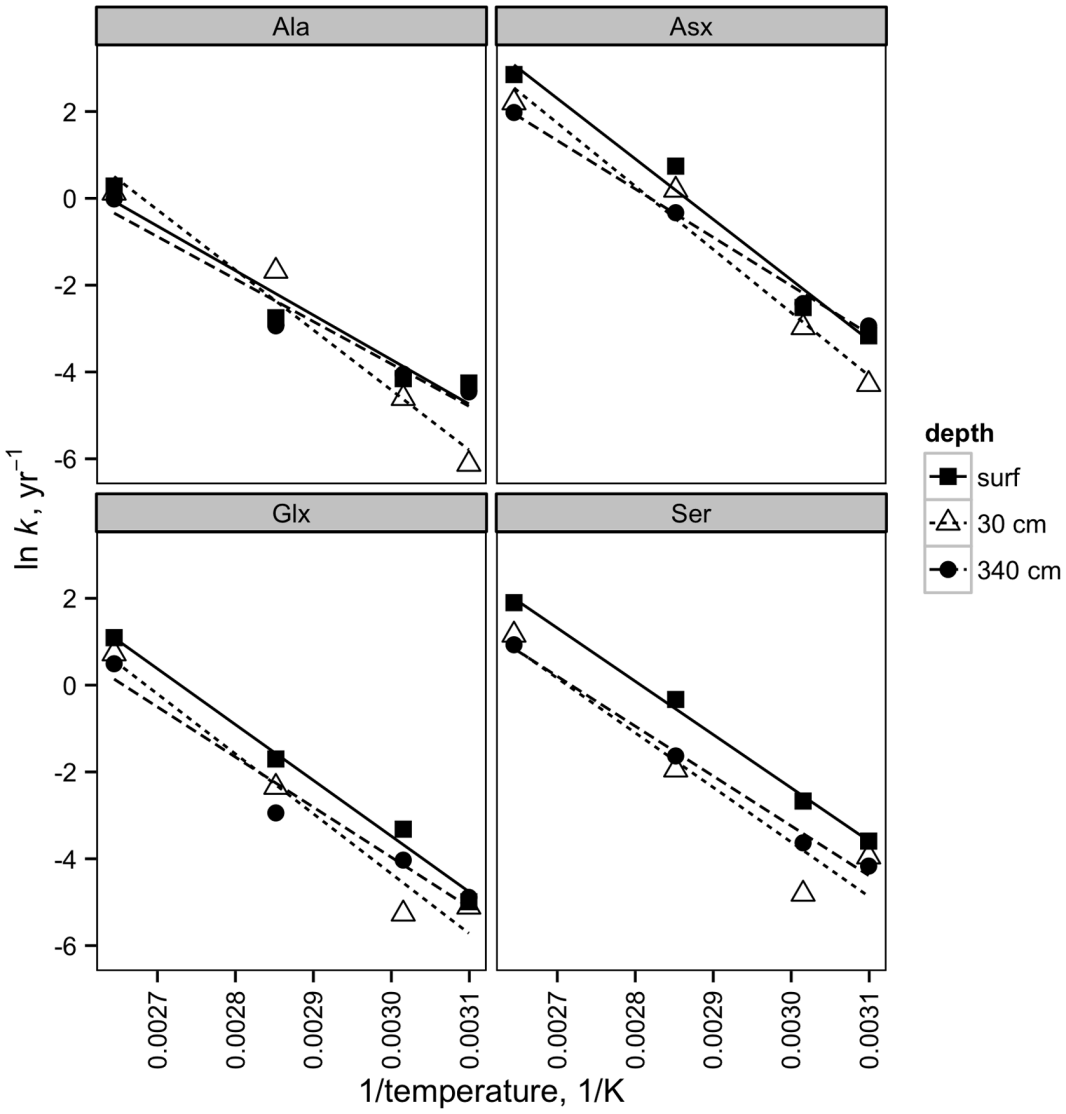
Arrhenius plot of measured racemization rate constants for each amino acid. Lines for each depth indicate the median rate constant predicted using the bootstrap-Monte Carlo approach (see Methods section for details).

Activation energies (i.e., the energy difference between the reactant and the transition state) were calculated using the slope of the curves in Fig. 2. Values were generally similar in surface and 30 cm sediment, and lower in 340-cm sediment (Table 2). Ala, which had a considerably lower activation energy in surface sediment than 30 cm sediment, is an exception. However, these differences were generally not distinguishable from error.

**Table 2:** Activation energies, E_a_, kJ mol^−1^; frequency factors (ln A), yr^−1^; and consensus Arrhenius equations for racemization rate constants (in which temperature T is in K and *k* is yr^−1^).

		Asx	Glx	Ser	Ala
E_a_	Surface	**118** (112, 124)	**102** (94, 115)	**103** (100, 107)	**86** (77, 96)
	30 cm	**121** (111, 136)	**111** (96, 131)	**104** (95, 115)	**108** (98, 122)
	340 cm	**95** (88, 104)	**98** (89, 110)	**98** (90, 110)	**83** (76, 90)
	consensus	**110**	**106**	**101**	**93**
ln(A)	Surface	**40.7** (38.7, 42.9)	**33.6** (30.8, 37.8)	**34.9** (33.8, 36.1)	**27.2** (24.4, 30.8)
	30 cm	**41.1** (37.8, 45.9)	**35.9** (31.1, 42.6)	**34.1** (31.1, 37.4)	**34.7** (31.6, 39.4)
	340 cm	**32.2** (29.8, 35.3)	**31.5** (28.5, 35.5)	**32.2** (29.4, 36.2)	**26.1** (23.7, 28.9)
	consensus	**37.5**	**34.4**	**33.3**	**30.0**
consensus	ln(k) =	37.5–1300/T	34.4–12700/T	33.3–12100/T	30.0–11100/T

Values in bold are the median of bootstrap-simulated values and values in parentheses indicate +/−1 standard deviation. Consensus values are based on observed rate constants at all sediment depth for each amino acid.

Racemization rate constants varied among amino acids and in some cases among depths (Table 1, Fig. 1). A wide range of chemical factors influences racemization rates [14]. One particularly important influence on racemization rates may be the extent of humification of amino acids in sediments: incorporation of amino acids into melanoidins is known to retard amino acid racemization [15]. pH also influences racemization rate constants, but racemization rate constants are not measurably affected by pH between pH 7 and 8.3 [26]. Since typical pH values in marine porewater are within this range, pH was unlikely to affect racemization rate constants. Changes in sediment mineralogy might also lead to variations in racemization rate constants since charged sediment surfaces could potentially stabilize racemization transitional states. Only little down-core variation in Aarhus Bay sediment mineralogy has been observed within the Holocene Marine Unit 2 sediment studied here [27]. Due to the difficulty of identifying the causes for variations in racemization rate constants among sediment horizons, we propose consensus equations for racemization rate constants as a function of temperature, based on the full set of observed rate constants for each amino acid, which should be applied to estimate racemization rate constants in the future (Table 3).

**Table 3:** Racemization at 3°C. Rate constants *k* are given in units of 10^−5^ yr^−1^.

		Asx	Glx	Ser	Ala
*k*	Surface	**2.3** (1.2, 4.0)	**1.7** (0.45, 4.0)	**4.3** (3.0, 6.0)	**4.1** (1.3, 9.5)
	30 cm	**0.92** (0.19, 2.6)	**0.41** (0.04, 2.0)	**1.2** (0.38, 3.0)	**0.50** (0.11, 1.3)
	340 cm	**11** (4.2, 24)	**1.3** (0.36, 3.3)	**2.5** (0.69, 5.6)	**4.8** (2.3, 8.7)
	Consensus	**3.2**	**0.73**	**2.5**	**1.7**
	Bada and Schroeder	**0.15**			**0.046**
*t_½_*	Surface	**15** (8.7, 28)	**21** (8.6, 80)	**8.0** (5.8, 11)	**8.4** (3.6, 27)
	30 cm	**38** (13, 180)	**85** (18, 780)	**30** (12, 90)	**70** (26, 329)
	340 cm	**3.1** (1.5, 8.3)	**26** (11, 96)	**14** (6.2, 50)	**7.3** (4.0, 15)
	Consensus	**11**	**47**	**14**	**20**
	Bada and Schroeder	**231**			**753**

Racemization halflives *t_½_* are given in units of 10^3^ yr. Values in bold are the median of bootstrap-simulated values and values in parenthesis indicate +/−1 standard deviation. Note that the Bada and Schroeder data [21] were measured using free amino acids in aqueous solution.

### Comparison to Previously Measured Racemization Rate Constants

**Figure 3:**
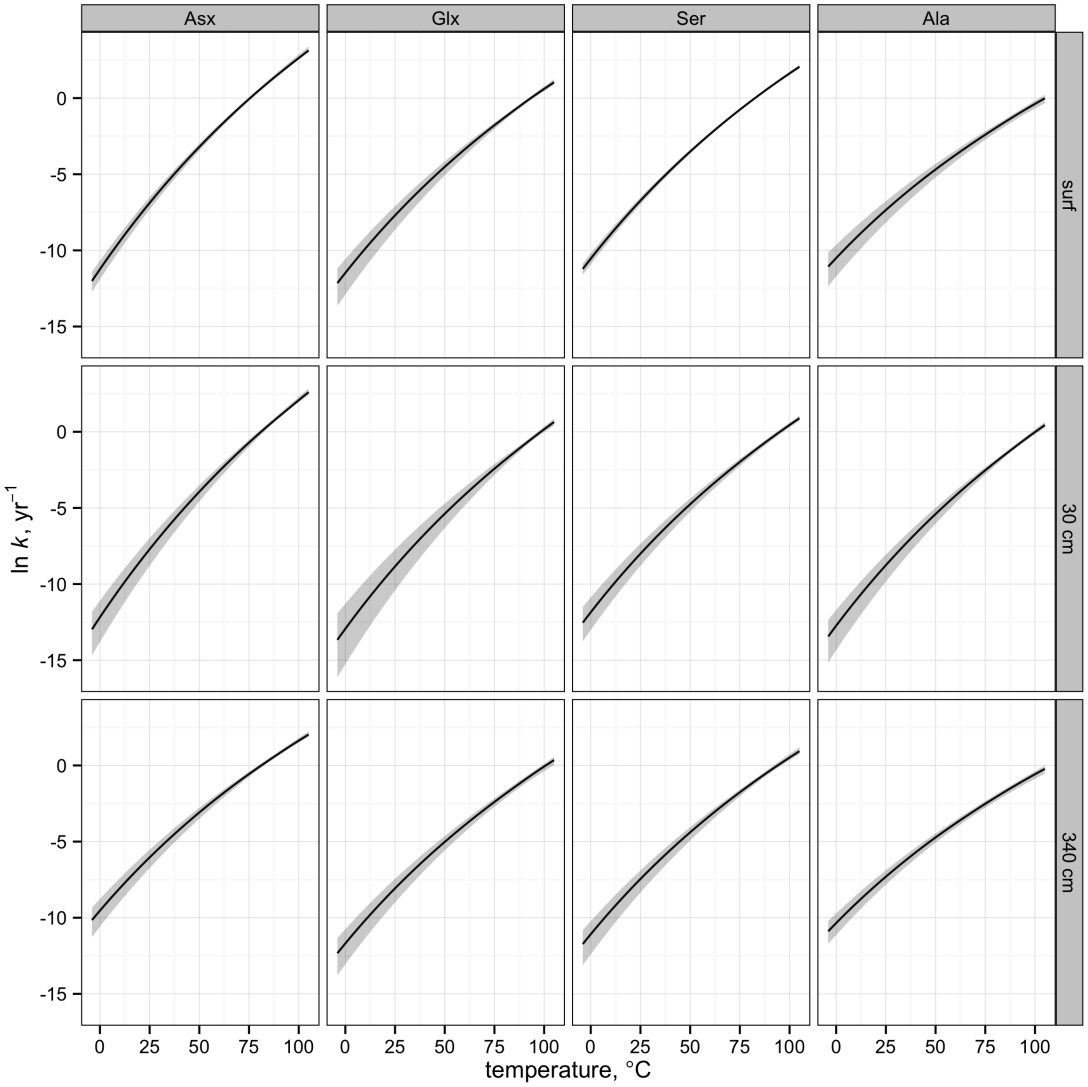
Simulated rate constants at −4–105°C. The solid line and shaded areas indicate predicted rate constant and error as a function of temperature (see Methods section for details).

Extrapolated to temperatures relevant to most marine sediment (we have arbitrarily chosen 3°C as a reference temperature), the rate constants measured in this study are considerably faster than those measured for free amino acids in aqueous solution by Bada [12] (Table 3; Fig. 3). The faster rates observed in this study are in accordance with observations that amino acids contained within proteins may racemize up to 7 times faster than the corresponding free amino acid [28].

The racemization rate constants and Arrhenius parameters measured here can also be compared to those measured in other biological media. By comparing observed d:l ratios to sediment age, Kvenvolden et al. [29] found rate constants of 1.5×10^−5^ yr^−1^ for Glu and 9.6×10^−6^ yr^−1^for Ala, although it is important to note that these rate constants were assessed in natural sediments with unknown temperature histories and with active biological processing of amino acids. Using a similar approach, Harada et al. [30] calculated lower racemization rate constants, 7.1–8.6×10^−6^ yr^−1^, for Asx in foraminifera picked from sediments. In the colonial anemone, *Gerardia* sp., the activation energy for Asx racemization was 122 kJ mol^−1^, with a predicted rate constant at 3°C of 3.2×10^−5^ yr^−1^, which is within the range of rate constants measured here [16].

Racemization kinetics have also been measured via heating experiments in a wide range of non-marine samples, for example in the heartwood of sequoia trees, with similar results: rate constants of 10.×10^−6^ yr^−1^ at 0°C, and activation energies close to 100 kJ mol^−1^, e.g. [31]. Harada et al [30] calculated lower racemization rate constants for Asx in foraminifera picked from sediments, in the range of 7.1–8.6×10^−6^ yr, by regressing the sediment age against observed d:l ratios. These lower rate constants may be related to higher contribution of contaminating d-amino acids from cell walls from bacteria harbored in younger sediments.

The results presented here highlight the problem of using amino acid enantiomeric ratios for geochronology, a practice which has been disputed for some time [32] but which remains in use [33]. The d:l amino acid ratios are indeed affected by microbial turnover of sediment organic matter and synthesis of new proteins in microbial biomass. Thus, it was shown in deep sediment cores from the Eastern Tropical Pacific Ocean that amino acids from plankton were broken down relatively early after burial and that the bulk of the amino acids in subsurface sediment was primarily derived from microbial necromass [7].

Amino acids in marine sediments appear to racemize at similar rates as those in a range of other environments. If biological activity were inhibited in the sediment, d:l ratios would be a useful tool for the age determination of buried organic matter. The ubiquity of microbial life in marine sediments, however, means that d:l ratios are always influenced by bacterial decomposition and by bacterially-derived organic matter in the sediment, as well as by abiotic racemization of L-amino acids. Accurate knowledge of abiotic racemization rate constants is therefore needed to use d:l ratios as a biomarker for the calculation of microbial biomass and necromass turnover times.

## Supporting Information

Table S1 **D:L values observed in the heating experiment.**
(XLSX)
