# Correction: Abiotic Racemization Kinetics of Amino Acids in Marine Sediments

**DOI:** 10.1371/journal.pone.0123837

**Published:** 2015-04-08

**Authors:** The PLOS ONE Staff

In Table 2, the row labeled “consensus” has two errors. One of the numbers is off by a factor of 10, and the values have been inappropriately rounded. Please view the correct Table 2 here.

**Table 2:** Activation energies, E_a_, kJ mol^−1^; frequency factors (ln A), yr^−1^; and consensus Arrhenius equations for racemization rate constants (in which temperature T is in K and *k* is yr^−1^).

		Asx	Glx	Ser	Ala
Ea	Surface	**118** (112, 124)	**102** (94, 115)	**103** (100, 107)	**86** (77, 96)
	30 cm	**121** (111, 136)	**111** (96, 131)	**104** (95, 115)	**108** (98, 122)
	340 cm	**95** (88, 104)	**98** (89, 110)	**98** (90, 110)	**83** (76, 90)
	consensus	**110**	**106**	**101**	**93**
ln(A)	Surface	**40.7** (38.7, 42.9)	**33.6** (30.8, 37.8)	**34.9** (33.8, 36.1)	**27.2** (24.4, 30.8)
	30 cm	**41.1** (37.8, 45.9)	**35.9** (31.1, 42.6)	**34.1** (31.1, 37.4)	**34.7** (31.6, 39.4)
	340 cm	**32.2** (29.8, 35.3)	**31.5** (28.5, 35.5)	**32.2** (29.4, 36.2)	**26.1** (23.7, 28.9)
	consensus	**37.5**	**34.4**	**33.3**	**30.0**
consensus	ln(k) =	37.48-13207/T	34.35-12752/T	33.32-12124/T	29.95-11304/T
